# Pharmacological Blockade of PPAR Isoforms Increases Conditioned Fear Responding in the Presence of Nociceptive Tone

**DOI:** 10.3390/molecules25041007

**Published:** 2020-02-24

**Authors:** Jessica C. Gaspar, Bright N. Okine, Alvaro Llorente-Berzal, Michelle Roche, David P. Finn

**Affiliations:** 1 Pharmacology and Therapeutics Department, National University of Ireland Galway, University Road, H91 W5P7 Galway, Ireland; jeccgaspar@gmail.com (J.C.G.); bnokine@gmail.com (B.N.O.); alvaro.llorente-berzal@nuigalway.ie (A.L.-B.); 2 Galway Neuroscience Centre, National University of Ireland Galway, University Road, H91 W5P7 Galway, Ireland; michelle.roche@nuigalway.ie; 3 Centre for Pain Research, National University of Ireland Galway, University Road, H91 W5P7 Galway, Ireland; 4 Physiology Department, National University of Ireland Galway, University Road, H91 W5P7 Galway, Ireland

**Keywords:** PPARs, anxiety, fear-conditioned analgesia, nociceptive behaviour, conditioned fear

## Abstract

Peroxisome proliferator-activated receptors (PPARs) are nuclear receptors with three isoforms (PPARα, PPARβ/δ, PPARγ) and can regulate pain, anxiety, and cognition. However, their role in conditioned fear and pain-fear interactions has not yet been investigated. Here, we investigated the effects of systemically administered PPAR antagonists on formalin-evoked nociceptive behaviour, fear-conditioned analgesia (FCA), and conditioned fear in the presence of nociceptive tone in rats. Twenty-three and a half hours following fear conditioning to context, male Sprague-Dawley rats received an intraplantar injection of formalin and intraperitoneal administration of vehicle, PPARα (GW6471), PPARβ/δ (GSK0660) or PPARγ (GW9662) antagonists, and 30 min later were re-exposed to the conditioning arena for 15 min. The PPAR antagonists did not alter nociceptive behaviour or fear-conditioned analgesia. The PPARα and PPARβ/δ antagonists prolonged context-induced freezing in the presence of nociceptive tone without affecting its initial expression. The PPARγ antagonist potentiated freezing over the entire trial. In conclusion, pharmacological blockade of PPARα and PPARβ/δ in the presence of formalin-evoked nociceptive tone, impaired short-term, within-trial fear-extinction in rats without affecting pain response, while blockade of PPARγ potentiated conditioned fear responding. These results suggest that endogenous signalling through these three PPAR isoforms may reduce the expression of conditioned fear in the presence of nociceptive tone.

## 1. Introduction

Peroxisome proliferator-activated receptors (PPARs) are ligand-dependent transcription factors and part of the nuclear hormone superfamily of receptors. There are three described isoforms: PPARα, PPARβ/δ and PPARγ [1].

All three isoforms are expressed in the brain and spinal cord [2]. Endogenous ligands at PPARs, include fatty acids [3], serotonin derivates [4], and *N*-acylethanolamines (NAEs) including anandamide (AEA) [5,6], *N*-palmitoylethanolamide (PEA) [7] and *N*-oleoylethanolamide (OEA) [8]. PPARs are involved in many physiological processes and are targets for currently in-use medicines for diabetes [9] and lowering cholesterol therapies [10]. Moreover, studies suggest that the PPAR signalling system may act as a modulator of pain [11], anxiety [12] and cognition [13,14,15].

PPARs are expressed in neural regions that play an important role in pain and fear/anxiety such as the prefrontal cortex (PFC) [2,16,17], hippocampus [2,12], amygdala [17], periaqueductal grey (PAG) [18], spinal cord [2], and dorsal root ganglion [19,20]. Previous studies have shown that the selective activation of PPARα [16,19,21], PPARβ/δ [22,23], and PPARγ [24,25] has antinociceptive effects. The administration of PEA [21,26] and OEA or OEA-derivated compounds [27,28,29] suppresses nociception.

Fear is well recognised to modulate pain responses. An example of this is the phenomenon known as fear-conditioned analgesia (FCA), in which exposure to a fearful stimulus suppresses nociception. Different neuromodulators are involved in FCA such as the opioid, GABAergic, glutamatergic, monoaminergic and endocannabinoid systems [30]. Recent studies have shown that levels of AEA, PEA and OEA, three endogenous ligands at PPARs, are increased in the basolateral amygdala of rats expressing FCA [31,32], suggesting a possible role for PPARs in this potent form of endogenous analgesia. In turn, pain can regulate fear responses. Post-traumatic stress disorder (PTSD) symptoms tend to be more pronounced in patients with chronic pain [33]. Additionally, patients with chronic pain are twice as likely to develop phobias [34]. There is some evidence that PPARγ blockade or knockout elicits anxiogenic effects in mice [12]. However, the role of PPARα or PPARβ/δ in modulating anxiety or fear responses remains unexplored. Furthermore, the role of PPARs in reciprocal interactions between pain and fear has not yet been investigated.

The aim of the present study was to investigate the hypothesis that endogenous signalling via PPARs modulates tonic inflammatory pain, fear and their interaction. We focused on the non-genomic, non-transcription-dependent effects of the PPAR isoforms, and while the genomic effects of PPARs on pain and fear-related behavior are also of interest and warrant investigation, they were outside the scope of the present manuscript. Specifically, we examined the effects of the administration of GW6471 (PPARα antagonist), GSK0660 (PPARβ/δ antagonist), and GW9662 (PPARγ antagonist) on formalin-induced nociceptive behaviour, fear-conditioned analgesia, and conditioned-fear related behaviour in rats.

## 2. Results

### 2.1. Systemic Administration of PPARα and PPARβ/δ Antagonists Had no Effect on Formalin-Evoked Nociceptive Behaviour or FCA

**Figure 1:**
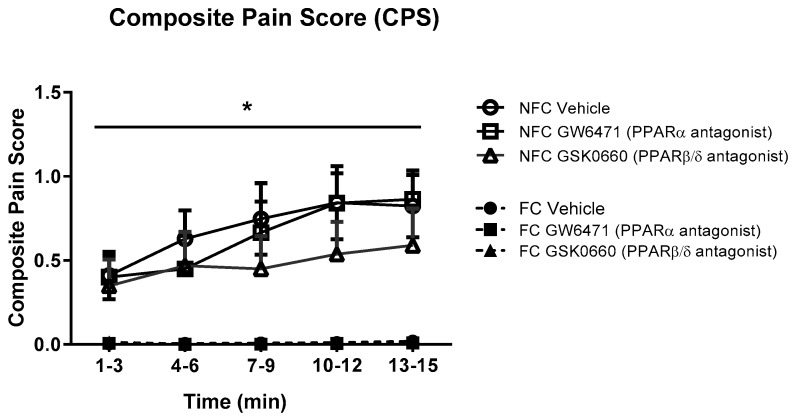
Effects of the systemic administration of selective PPARα and PPARβ/δ antagonists on formalin-evoked nociceptive behaviour in non-fear conditioned (NFC) and fear conditioned (FC) rats. Composite pain score was calculated as (pain 1 + 2 × [pain 2])/total duration of analysis period (see Materials and Methods for further information). Data (mean ± S.E.M) are represented in 3-min time bins (*n* = nine rats per group). According to a repeated measures ANOVA (*p* < 0.05), * significant main effect of fear conditioning.

**Figure 2:**
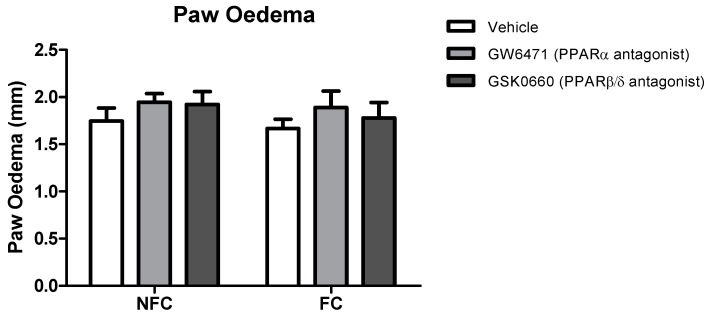
Effects of the systemic administration of selective PPARα and PPARβ/δ antagonists on formalin-evoked hind paw oedema in non-fear conditioned (NFC) and fear conditioned (FC) rats. Paw oedema was assessed by measuring the change in the diameter of the right hind paw immediately before, and 45 min after, formalin administration. Data are expressed as mean ± S.E.M, *n* = nine rats per group.

The intraplantar administration of formalin into the right hind paw produced robust nociceptive behaviour as evidenced by the composite pain score (CPS). Fear-conditioned rats exhibited significantly reduced pain-related behaviour throughout the trial, confirming expression of FCA [F_1, 48_ = 38.104, *p* < 0.05]. Formalin-evoked nociceptive behaviour was unaltered by systemic administration of GW6471 (PPARα antagonist) or GSK0660 (PPARβ/δ antagonist) in both non-fear-conditioned (NFC) and fear-conditioned (FC) rats (Figure 1). Similar analysis using 2-way ANOVA revealed no significant effect of fear-conditioning or PPAR antagonists on formalin-induced paw oedema (Figure 2).

### 2.2. Systemic Administration of PPARα and PPARβ/δ Antagonists Prolongs Fear-Related Behaviour in Formalin-Treated Rats

**Figure 3:**
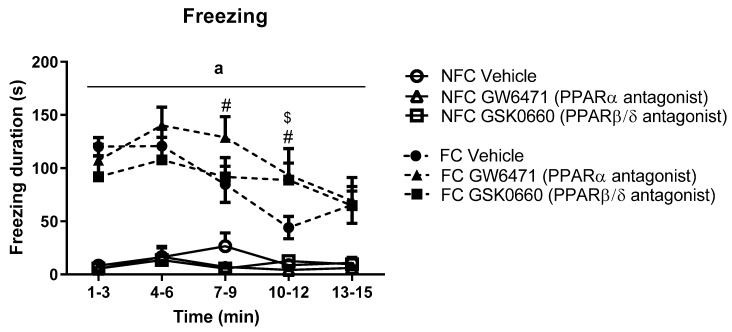
Temporal profile of the effects of fear conditioning and systemic administration of selective PPARα and PPARβ/δ antagonists on freezing in non-fear conditioned (NFC) and fear conditioned (FC) rats. Post hoc analysis with Student Newman-Keuls revealed that all formalin-injected FC groups exhibited significantly greater duration of freezing compared with NFC counterparts (^a^ *p* < 0.001). Treatment with GW6471 in FC rats significantly increased freezing duration in two of the 3-min time bins (# *p* < 0.05, vs. FC Vehicle), and treatment with GSK0660 significantly increased freezing duration in one of the 3-min time bins ($ *p* < 0.05, vs. FC Vehicle). Data are expressed as mean ± S.E.M (*n* = 7–9 per group).

Repeated measures ANOVA revealed a significant effect of fear conditioning (F_1, 46_ = 80.397, ^a^ *p* < 0.05), time (F_2.871, 132.072_ = 7.213, *p* < 0.001), and fear conditioning x time (F_3.415, 132.072_ = 5.961, *p* <.001) on freezing duration. Post hoc analysis indicated that FC vehicle-treated rats exhibited significantly increased freezing duration in the early part of the trial (from t_1–3_ to t_4–6_) compared with NFC counterparts (Figure 3). Systemic administration of GW6471 (# *p* < 0.05, vs. FC Vehicle) or GSK0660 ($ *p* < 0.05, vs. FC Vehicle) prolonged expression of contextually induced freezing (see Figure 3).

### 2.3. Systemic Administration of PPARα and PPARβ/δ Antagonists Had no Effect on General/Motor Behaviour

**Figure 4:**
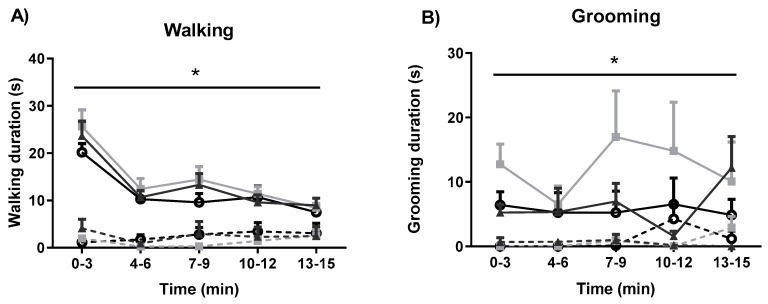

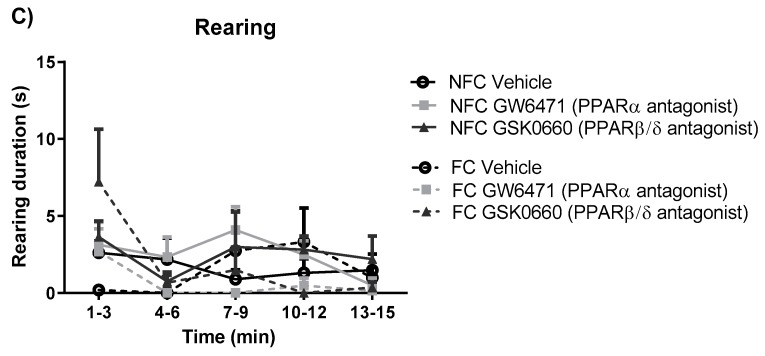
Effects of fear-conditioning and systemic administration of selective PPARα and PPARβ/δ antagonists on walking duration (**A**), grooming duration (**B**), and rearing duration (**C**). Data (mean ± S.E.M) are represented in 3-min time bins (*n* = nine rats per group). According to a repeated measured ANOVA (*p* < 0.05), * significant main effect of fear conditioning.

The effects of fear-conditioning and systemic administration of GW6471 and GSK0660 on motor behaviour were also assessed (Figure 4). Fear conditioning resulted in decreased walking in all groups (F_1, 48_ = 110.009, *p* < 0.05). PPAR antagonists did not induce any significant effect on walking in either NFC or FC rats (Figure 4A). Fear conditioning decreased total grooming duration (F_1, 45_ = 39.01, *p* < 0.05), an effect not significantly altered by PPAR antagonists (Figure 4B). Neither fear conditioning nor antagonist treatment had any effect on total rearing duration (Figure 4C).

### 2.4. Systemic Administration of PPARα Antagonist Has no Effect on Formalin-Evoked Nociceptive Behaviour or FCA

**Figure 5:**
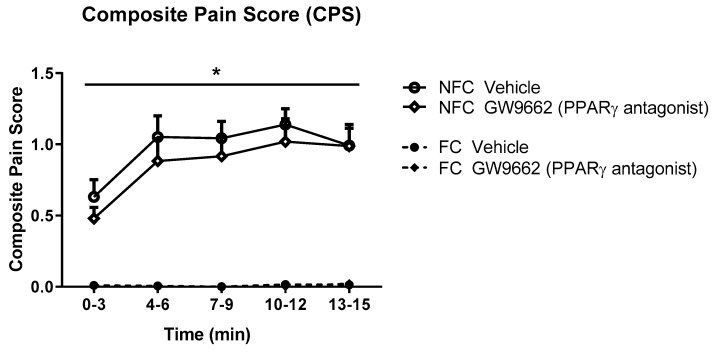
Effects of systemic administration of selective PPARγ antagonist on formalin-evoked nociceptive behaviour in non-fear conditioned (NFC) and fear conditioned (FC) rats. Composite pain score was calculated as (pain 1 + 2 × [pain 2])/total duration of analysis period. Data (mean ± S.E.M) are represented in 3-min time bins (*n* = nine rats per group). According to a repeated measured ANOVA (*p* < 0.05), * significant main effect of fear conditioning.

**Figure 6:**
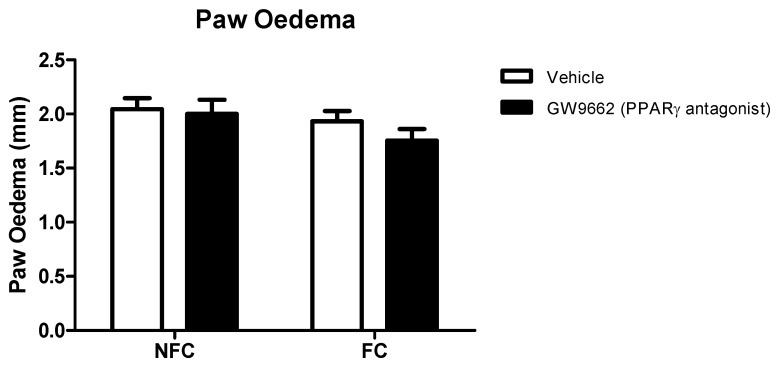
Effects of systemic administration of selective PPARγ antagonist on formalin-evoked hind paw oedema in non-fear conditioned (NFC) and fear conditioned (FC) rats. Paw oedema was assessed by measuring the change in the diameter of the right hind paw immediately before, and 45 min after, formalin administration. Data are expressed as mean ± S.E.M, *n* = nine rats per group.

As with Experiment 1, the intra-plantar injection of formalin resulted in robust nociceptive behaviour as indicated by the CPS (Figure 5). Repeated measures ANOVA revealed a significant effect of fear conditioning (F_1,32_ = 128.8, *p* < 0.05), but not drug treatment, on formalin-evoked nociceptive behaviour. Neither fear conditioning nor GW9662 administration had any significant effect on the formalin-induced change in hind paw diameter (oedema) (Figure 6).

### 2.5. Systemic Administration of PPARγ Antagonist (GW9662) Enhances Fear-Related Behaviour in Formalin-Treated Rats

**Figure 7:**
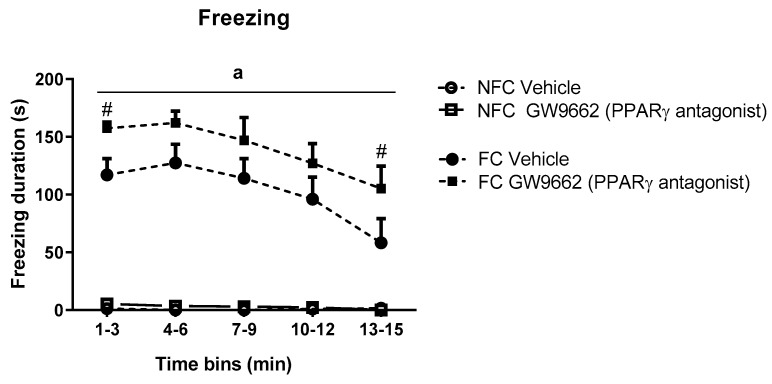
Temporal profile of the effects of systemic administration of selective PPARγ antagonist on freezing duration in NFC and FC rats. Two-way ANOVA revealed a significant effect of conditioning (^a^ *p* < 0.001) on freezing duration. Post hoc analysis revealed that treatment with GW9662 in FC rats significantly affected freezing duration in two time bins (# *p* < 0.05, vs. FC Vehicle). Data are expressed as 3 min bins (mean ± S.E.M, *n* = nine rats per group).

Repeated measures ANOVA revealed significant effects of conditioning (F_1,32_ = 184.373, ^a^ *p* < 0.05), drug treatment (F_1,32_ = 4.952, *p* < 0.05), time (F_2.587, 82.768_ = 8.754, *p* < 0.05), and a time x conditioning effect (F_2.587, 82.768_ = 7.978, *p* < 0.05) on the duration of freezing behaviour. Post hoc analysis with Student Newman-Keuls test indicated that both groups of fear-conditioned rats exhibited significantly increased freezing duration over the entire trial compared with non-fear-conditioned counterparts and that systemic administration of GW9662 potentiated contextually induced freezing behaviour over the course of the trial (# *p* < 0.05 vs FC Vehicle) (Figure 7).

### 2.6. Systemic Administration of PPARγ Antagonist (GW9662) Had no Effects on General/Motor Behaviour

**Figure 8:**
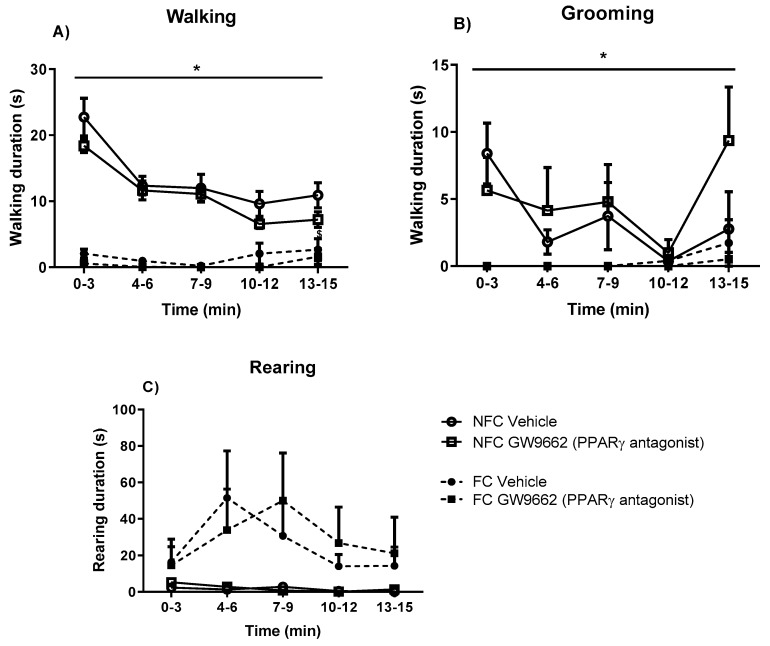
Effects of fear-conditioning and systemic administration of selective PPARγ antagonist on walking duration (**A**), grooming duration (**B**), and rearing duration (**C**). Data (mean ± S.E.M) is represented in 3-min time bins (*n* = nine rats per group). According to a repeated measured ANOVA (*p* < 0.05), * significant overall effect of fear conditioning.

Repeated measures ANOVA revealed significant effects of fear conditioning but not drug treatment on duration of walking (F_1, 32_ = 137.809, * *p* < 0.05) and grooming (F_1, 32_ = 31.7, * *p* < 0.05) (Figure 8). Neither drug treatment nor fear conditioning had any effects on rearing duration.

## 3. Discussion

The present study is, to our knowledge, the first to investigate the role of all three PPAR isoforms in reciprocal interactions between pain and fear. Systemic administration of the PPARα and PPARβ/δ antagonists to rats prolonged context-induced freezing in the presence of formalin-evoked nociceptive tone without affecting its initial expression, while the PPARγ antagonist potentiated freezing expression over the entire trial. These effects on fear-related behaviour were observed in the absence of any effects on formalin-evoked nociceptive behaviour or locomotor activity. These novel data suggest that the pharmacological blockade of PPARα and PPARβ/δ in the presence of formalin-evoked nociceptive tone, impaired short-term, within-trial fear-extinction in rats without affecting pain response, while the pharmacological blockade of PPARγ potentiated conditioned fear-related behaviour. Thus, endogenous signalling through these three PPAR isoforms may serve to reduce the expression of conditioned fear in the presence of nociceptive tone.

The data herein suggest a modulatory role for PPARs in fear-related behaviour in the presence of nociceptive tone. We propose that the blockade of PPARα and PPARβ/δ delayed the short-term, within-trial extinction of fear memory without affecting initial expression of fear-related behaviour. Extinction is defined as a learned inhibition of retrieval of previously acquired memories. Therefore, the blockade of PPARα and PPARβ/δ may be impairing the formation of a new memory upon re-exposure to the conditioned arena. Most studies investigating the role of PPARs in memory have investigated their role in models of mnemonic impairment, such as diabetes-induced cognitive dysfunction [35,36], morphine-induced mnemonic dysfunction [13], scopolamine-induced memory impairment [37,38,39] and others [40,41,42,43]. There are studies showing that the modulation of PPARs may also affect memory in subjects whose mnemonic abilities were preserved. For instance, Mazzola et al [14] have shown that the intraperitoneal administration of WY14643, a PPARα synthetic agonist, enhanced memory acquisition. Campolongo et al. [44] have shown that post-training administration of the endogenous PPAR ligand OEA enhanced memory consolidation in both spatial and passive-avoidance learning tests, effects that were abolished in mutant mice lacking PPARα. On the other hand, Varvel et al [45] demonstrated that the administration of OEA and PEA before testing did not have any effect on working memory. A potential alternative explanation for our findings is that the blockade of PPARα and PPARβ/δ enhanced the recall of fearful memories, however the lack of effect of the PPARα and PPARβ/δ antagonists on the initial expression of freezing upon re-exposure to the context argues against this. In contrast, we found that the systemic administration of the PPARγ antagonist potentiated the expression of initial freezing upon context re-exposure, and that this potentiation was maintained over the entire trial. Thus, it is possible that blockade of PPARγ enhances fear memory recall, or is in itself pro-aversive (i.e., supporting an anxiolytic effect of PPARγ signalling). The latter interpretation may be more likely because previous studies demonstrated that the PPARγ activation rather than blockade improves mnemonic performance. For example, Gemma et al. [46] have shown that the oral administration of rosiglitazone, a PPARγ agonist, improved cognitive performance in aged rats compared to young controls exposed to contextual fear conditioning. Similarly, Babaei et al. [13] have shown that pioglitazone, another PPARγ agonist, improved the performance of mice with mnemonic impairment induced by morphine. Other studies have shown improved cognitive performance in pioglitazone-treated animals [38,40,42,43,47]. Further evidence in support of an anxiolytic effect of PPARγ signalling comes from recent work by Youssef et al. [48], demonstrating that the administration of a PPARγ antagonist blocked the anxiolytic effect of beta-caryophyllene. Additionally, repeated stress decreased PPARγ expression in the amygdala, and treatment with buspirone or minocycline, two drugs having anxiolytic effects, recovered PPARγ expression in the same region [49]. Furthermore, PPARγ blockade or knockout was shown to have anxiogenic effects in mice [12]. These studies, together with our data herein, suggest an anti-aversive/anxiolytic effect of PPARγ signalling.

Our results suggest that endogenous signalling at PPARα, PPARβ/δ and PPARγ does not mediate or modulate formalin-evoked nociceptive behaviour. Our findings are in accordance with Donvito et al. [50] who demonstrated that intraperitoneal administration of the PPARα antagonist GW6471 did not affect formalin-evoked nociceptive behaviour in mice. Previous reports have shown that systemic administration of PPARα [25,28,51] and PPARβ/δ [52] agonists attenuated acute inflammatory pain behaviour, which indicates an antinociceptive effect of PPARα and PPARβ/δ activation by exogenously administered agonists (for review, see [11]). However, less is known about the effects of the blockade of these receptors on inflammatory pain. To our knowledge, the current study is the first to investigate the effects of the blockade of PPARβ/δ on inflammatory pain. Previous studies have shown that systemic administration of pioglitazone, a widely used PPARγ agonist, attenuates formalin-induced nociceptive response [24,25]. In their study, Mansouri et al. [24] also indicated that systemic administration of GW9662 alone did not have any effect on nociceptive behaviour, which is in line with our findings. A limitation of our study is that the design and timeline of our experiments was such that we did not investigate potential genomic effects of PPARs, but instead focused on the non-genomic, non-transcription-dependent effects of the PPAR isoforms on pain and fear-related behavior. Future studies should investigate whether PPARs modulate pain and pain-fear interactions via effects at the genomic/transcriptional level.

FCA is a potent suppression of nociceptive responses upon exposure to a fearful stimulus. It has been shown to be associated with increased levels of AEA, an endocannabinoid which also binds to PPARs, in the basolateral amygdala (BLA) [32] and in the dorsolateral periaqueductal grey (dlPAG) [31] and a strong trend for increased tissue levels of PEA and OEA, endogenous ligands of PPARs, in the BLA [7,8]. The present study investigated the effects of administration of PPAR antagonists on FCA. The data herein demonstrate that fear conditioning profoundly reduces formalin-evoked nociceptive behaviour via FCA as we and others have shown previously [31,53,54,55], and that the blockade of PPARα, PPARβ/δ or PPARγ does not affect expression of FCA. However, a limitation of the method chosen in our experiments is that the trial duration (15 min) is short and, consequently, restricts an analysis of possible alterations in FCA at later time points beyond the initial 15 min period where FCA is very robust. Specifically, an enhancement of FCA by PPARs blockade would have been difficult to observe due to the minimal expression of nociceptive behaviour in FC rats during this initial 15 min period. However, we hypothesized that the PPAR antagonists would attenuate FCA, and previous work has demonstrated that it is possible to completely attenuate/prevent maximal or near maximal FCA in rats in our FCA paradigm with systemic administration of antagonists (e.g., the CB_1_ receptor antagonist rimonabant; [56]). So, we do believe that if PPARs were mediating FCA we would have seen at least a partial attenuation of this FCA in the present studies. Future investigations using an extended trial duration could be of value in further exploring the role of these receptors in FCA.

## 4. Materials and Methods

### 4.1. Animals

Adult male Sprague-Dawley rats (total *n* = 90, 260–280 g) were obtained from Envigo (Huntingdon, Cambridgeshire, United Kingdom). Animals from Experiment 1 and 2 were housed in groups of 3 upon arrival, and singly housed after 3 days for the duration of the study. The animals were maintained at 22 ± 2 °C under standard lighting conditions (12:12 h, lights on from 07:00 to 19:00). All experiments were conducted during the light phase and food (14% HarlanTeklad2014 Maintenance Diet, Envigo, Huntingdon, Cambridgeshire, United Kingdom) and water were available ad libitum. The experimental procedures were approved by the Animal Care and Research Ethics Committee, National University of Ireland Galway. The work was carried out under license from the Health Products Regulatory Authority in the Republic of Ireland and in accordance with EU Directive 2010/63.

### 4.2. Drugs

The PPARα antagonist, GW6471, PPARβ/δ antagonist, GSK0660, and PPARγ antagonist, GW9662 (all obtained from Tocris Bioscience, Bristol, UK) were dissolved in a 1:1:8 (ethanol, cremophor; saline 0.9%) vehicle solution. The dose of GW6471 (2 mg/kg) was chosen based on studies in the literature demonstrating its efficacy in reversing PEA-induced neuroprotective effects [57]. The dose of GSK0660 (1 mg/kg) was also chosen based on previous studies [58,59]. Likewise, the dose of GW9662 (2 mg/kg)) was chosen based on the studies of Griggs et al. [60] and Morgenweck et al. [61] showing that this dose was effective in reversing pioglitazone’s analgesic effects. In addition, we have recently generated data (unpublished) indicating that the single acute administration of the three PPAR antagonists at the doses used herein has no effects in the elevated plus maze, open-field or light-dark box tests of anxiety. Formalin (2.5%) was prepared from a 37% stock solution (Sigma-Aldrich, Dublin, Ireland) diluted in 0.9% sterile saline.

### 4.3. Experimental Design

**Figure 9:**
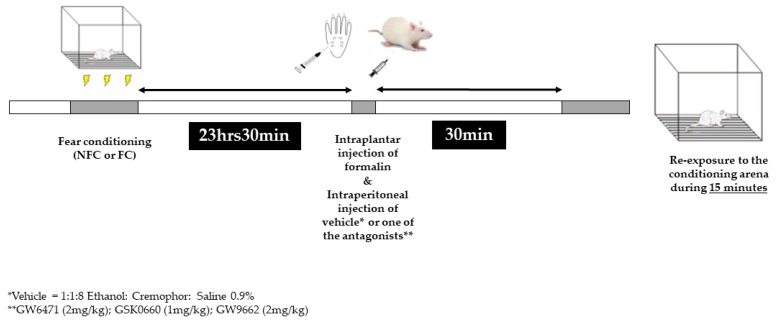
Graphical representation of the experimental design.

The FCA paradigm was essentially as described in our previous studies [56,62,63]. Briefly, there were two phases: conditioning (day 1) and test (day 2). On the conditioning day, rats were placed in a perspex chamber (30 cm × 30 cm × 30 cm) and after 15 s they received the first of 10 footshocks (0.4 mA, 1 s duration, LE85XCT Programmer and Scrambled Shock Generator; Linton Instrumentation, Norfolk, UK) spaced by 60 s. Control animals were placed in the chamber for an equivalent time (10 min) without receiving any footshock. Animals were randomly assigned to one of 6 groups—rats that received footshocks (FC) or no footshocks (NFC) treated with (experiment 1) PPARα antagonist GW6471, PPARβ/δ antagonist GSK0660 or vehicle (1:1:8, ethanol: cremophor: saline 0.9%) or in experiment 2 PPARγ antagonist GW9662 or vehicle (1:1:8, ethanol: cremophor: saline 0.9%). The test day started 23 h 30min after the end of the conditioning phase. First, the rats received a 50 µL injection of formalin (2.5% in 0.9% saline) into the right hind paw under brief isoflurane anaesthesia (3% in O_2_; 0.8 L·min^−1^). Immediately after, still under anaesthesia, the animals in Experiment 1 received an intraperitoneal administration of either the PPARα antagonist GW6471, the PPARβ/δ antagonist GSK0660 or vehicle (volume of injection 3 mL/kg), and animals in Experiment 2 received an intraperitoneal injection of either PPARγ antagonist (GW9662) or vehicle. Thirty minutes later, or 24 h after footshock, the rats were re-exposed to the conditioning chamber (Figure 9). A video camera located beneath the observation chamber was used to monitor animal behaviour for 15 min. A 15 min duration re-exposure was chosen on the basis of previous studies from our group demonstrating that fear-induced analgesia peaks within this time period [31,54,64]. Therefore, if PPARs are mediating FCA, we would expect to observe partial attenuation at this time point in the present studies. In both experiments, rats from each of the different groups were tested in a pseudorandomised sequence in order to control for order of testing. At the end of the test phase (45 min post-formalin injection), rats were killed by decapitation. Formalin-induced oedema was assessed by measuring the change in the diameter of the right hind paw immediately before, and 45 min after, formalin administration, using Vernier callipers.

### 4.4. Behavioural Analysis

Behaviour was analysed using EthoVision 11.5XT software (Noldus Information Technology, Wageningen, The Netherlands), which allowed for continuous event recording over each 15-min trial. A trained observer, blind to the experimental conditions, rated formalin-evoked nociceptive behaviour according to the weighted composite pain scoring technique [65]. According to this method, pain behaviours are categorized as time spent raising the right hindpaw above the floor without contact with any other surface (pain 1) and time spent holding, licking, biting, shaking or flinching the paw (pain 2) to obtain a composite pain score (CPS). CPS was calculated as (pain 1 + 2 × [pain 2])/total duration of analysis period. Duration of freezing (defined as the cessation of all visible movement except that necessary for breathing) was also assessed as an index of fear-related behaviour. Additionally, the duration of general behaviours including walking, grooming, and rearing were also measured.

### 4.5. Statistical Analysis

SPSS 22.0 statistical package was used to analyse all data. Normality and homogeneity of variance were assessed using Shapiro–Wilk and Levene tests, respectively. Behavioural data were analysed by 2-way ANOVA or repeated measures ANOVA (when applicable) followed by Student Newman–Keuls post hoc test. Data are expressed as group means ± standard error of the mean (SEM) and were considered significant when *p* < 0.05.

## 5. Conclusions

In conclusion, this study has shown that the systemic administration of PPARα and PPARβ/δ impaired fear-extinction while PPARγ antagonism enhances conditioned fear-related behaviour in rats exposed to an inflammatory pain stimulus. These effects on fear-related behaviour were independent of effects on pain-related behaviour, and thus indicate a possible modulatory role for PPARs in fear/anxiety expression. Further investigations are necessary to elucidate the molecular mechanisms involved in this modulation. The present study contributes to our understanding of the role of PPARs in pain–fear interactions. Furthermore, the study provides preclinical evidence that PPARs regulate fear responses in the presence of pain, laying the foundation for further preclinical and clinical translational studies towards the development of novel therapies for comorbid pain and fear-related disorders.
